# Nitrogen fixation in eukaryotes – New models for symbiosis

**DOI:** 10.1186/1471-2148-7-55

**Published:** 2007-04-04

**Authors:** Christoph Kneip, Peter Lockhart, Christine Voß, Uwe-G Maier

**Affiliations:** 1 Department of Cell Biology, Philipps-University Marburg, Marburg, Germany; 2 Department of Molecular Biology, Max-Planck-Institute for Infection Biology, Berlin, Germany; 3 Allan Wilson Centre for Molecular Ecology and Evolution, Institute of Molecular BioSciences, Massey University, Palmerston North, New Zealand

## Abstract

**Background:**

Nitrogen, a component of many bio-molecules, is essential for growth and development of all organisms. Most nitrogen exists in the atmosphere, and utilisation of this source is important as a means of avoiding nitrogen starvation. However, the ability to fix atmospheric nitrogen via the nitrogenase enzyme complex is restricted to some bacteria. Eukaryotic organisms are only able to obtain fixed nitrogen through their symbiotic interactions with nitrogen-fixing prokaryotes. These symbioses involve a variety of host organisms, including animals, plants, fungi and protists.

**Results:**

We have compared the morphological, physiological and molecular characteristics of nitrogen fixing symbiotic associations of bacteria and their diverse hosts. Special features of the interaction, e.g. vertical transmission of symbionts, grade of dependency of partners and physiological modifications have been considered in terms of extent of co-evolution and adaptation. Our findings are that, despite many adaptations enabling a beneficial partnership, most symbioses for molecular nitrogen fixation involve facultative interactions. However, some interactions, among them endosymbioses between cyanobacteria and diatoms, show characteristics that reveal a more obligate status of co-evolution.

**Conclusion:**

Our review emphasises that molecular nitrogen fixation, a driving force for interactions and co-evolution of different species, is a widespread phenomenon involving many different organisms and ecosystems. The diverse grades of symbioses, ranging from loose associations to highly specific intracellular interactions, might themselves reflect the range of potential evolutionary fates for symbiotic partnerships. These include the extreme evolutionary modifications and adaptations that have accompanied the formation of organelles in eukaryotic cells: plastids and mitochondria. However, age and extensive adaptation of plastids and mitochondria complicate the investigation of processes involved in the transition of symbionts to organelles. Extant lineages of symbiotic associations for nitrogen fixation show diverse grades of adaptation and co-evolution, thereby representing different stages of symbiont-host interaction. In particular cyanobacterial associations with protists, like the *Rhopalodia gibba*-spheroid body symbiosis, could serve as important model systems for the investigation of the complex mechanisms underlying organelle evolution.

## Background

Historically, the phenomenon of symbiosis has been defined as a close and prolonged interaction between two different species [1]. This includes parasitic, mutualistic and commensalistic interactions. However, more modern interpretations use the term "symbiosis" for interactions, which are more or less beneficial for both partners. Here, we use the term "mutualistic symbiosis" or "mutualism" for symbiotic interactions where a mutual benefit is confirmed. For interactions in general and where the exact nature of interaction is unknown or is not easily defined, we use the general term of "symbiosis".

It is generally thought that all eukaryotic organisms are descendents of progenitors in which at least two partners have interacted symbiotically. Mitochondria have originated from an α-proteobacterial ancestor, which was dramatically reduced during evolution [2,3]. Plastids, the typical organelles of photoautotrophic eukaryotes, are thought to have been derived from the merger of a cyanobacterial-like progenitor and a phagotrophic eukaryote [4]. The driving force for the close interactions that have led to organelle formation appear to be the metabolic needs of at least one of the participants in the interaction. In the case of mitochondria, ATP synthesis carried out by the α-proteobacterial symbiont has been the principal driving force for the co-evolution of both partners. In the case of plastids, the need for photosynthetic products has presumably driven symbiosis. Both metabolic capacities are exclusively prokaryotic inventions and only symbiotic interaction has allowed them to be used by eukaryotes. Prokaryotic invention and eukaryotic utilisation through symbiosis also applies to molecular nitrogen fixation. Nitrogen is an essential compound of many molecules, including proteins, nucleic acids and vitamins. Associations of eukaryotic host organisms with nitrogen-fixing bacteria occur in many environments and have thus increased the bioavailability of nitrogen. These associations are numerous and diverse, ranging from loose interactions to highly regulated intracellular symbioses.

Here we compare the morphological, physiological and molecular characteristics of symbiotic nitrogen fixing bacteria and their host organisms (animals, fungi, plants and protists). We classify the evolutionary state of some of these interactions, and discuss the potential of these for becoming model systems for investigating the molecular basis of the transition from endosymbiont to organelle [5,6].

### Molecular nitrogen fixation and nitrogenase

Most animals and fungi use nutrition to heterotrophically acquire nitrogen bound in biomolecules. However, other organisms including plants and many bacteria use inorganic nitrogen compounds like ammonium or nitrate bound to soil or present in water. The fixation of molecular nitrogen into bioavailable compounds for cellular anabolism is a process restricted to some bacteria. Such bacteria are termed diazotrophs, as they obtain all their nitrogen by fixing molecular nitrogen.

**Figure 1:**
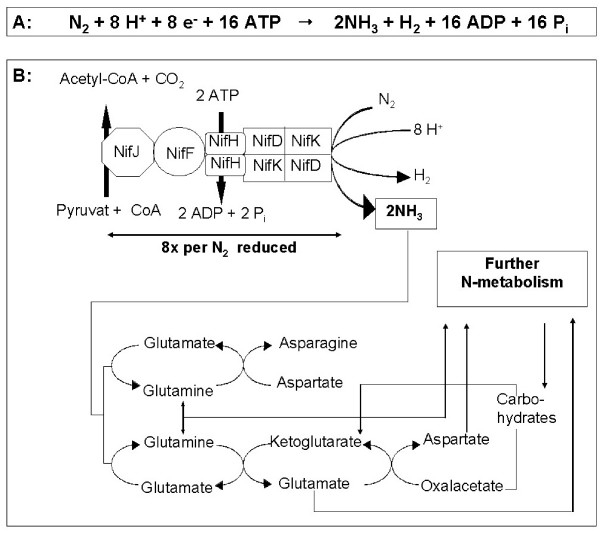
**Reaction and molecular mechanism of biological nitrogen fixation**. **A**. General reaction of molecular nitrogen fixation **B**. Schematic structure and operation of the nitrogenase enzyme complex and subsequent metabolism of nitrogen. Electrons are transferred from reduced ferredoxin (or flavodoxin) via azoferredoxin to molybdoferredoxin. Each mol of fixed nitrogen requires 16 mol ATP hydrolyzed by the NifH protein. The NH_3_ produced is utilised in the synthesis of glutamine or glutamate, respectively, for N-metabolism. NifJ: pyruvate flavodoxin/ferrodoxin oxidoreductase, NifF: Flavodoxin/Ferredoxin).

During biological nitrogen fixation (BNF) molecular nitrogen is reduced (Figure 1A) in multiple electron transfer reactions, resulting in the synthesis of ammonia and the release of hydrogen [7]. Ammonium is then used for the subsequent synthesis of biomolecules. This reduction of molecular nitrogen to ammonium is catalyzed in all nitrogen-fixing organisms via the nitrogenase enzyme complex in an ATP-dependent, highly energy consuming reaction (Figure 1B). The nitrogenase complex is comprised of two main functional subunits, dinitrogenase reductase (azoferredoxin) and dinitrogenase (molybdoferredoxin) [8]. The structural components of these subunits are the Nif (nitrogen fixation) proteins NifH (γ_2 _homodimeric azoferredoxin) and NifD/K (α_2_β_2 _heterotetrameric molybdoferredoxin). Basically three types of nitrogenases are known based on the composition of their metal centres: iron and molybdenum (Fe/Mo), iron and vanadium (Fe/V) or iron only (Fe) [9]. The most common form is the Fe/Mo-type found in cyanobacteria and rhizobia. An important feature of the nitrogenase enzyme complex is its extreme sensitivity to even minor concentrations of oxygen. In aerobic environments and in photoautotrophic cyanobacteria, where oxygen is produced in the light reactions of photosynthesis [10], nitrogenase activity must be protected. This protection is realised by different mechanisms in nitrogen fixing bacteria, depending on their cellular and physiologic constitutions. Aerobic bacteria like *Azotobacter *limit high intracellular oxygen concentrations by high rates of respiratory metabolism in combination with extracellular polysaccharides to reduce oxygen influx [11,12]. In some filamentous cyanobacteria, BNF is restricted to specialised cells, the heterocysts, which are separated from other cells, and show reduced photosynthetic activity without oxygen production [13,14]. Unicellular cyanobacteria combine photosynthesis and nitrogen fixation within the same cell and show a temporary separation of these two pathways where BNF is restricted to the dark period, when the oxygen-levels are low [15]. In addition to these protections, the concentration of oxygen can be decreased by biochemical pathways like the Mehler-reaction or by special oxygen-scavenging molecules such as cyanoglobin and leghemoglobin, the latter playing a major role in rhizobia-plant interactions [16,17].

### Diversity and specificity of symbioses between nitrogen fixing bacteria and eukaryotes

**Figure 2:**
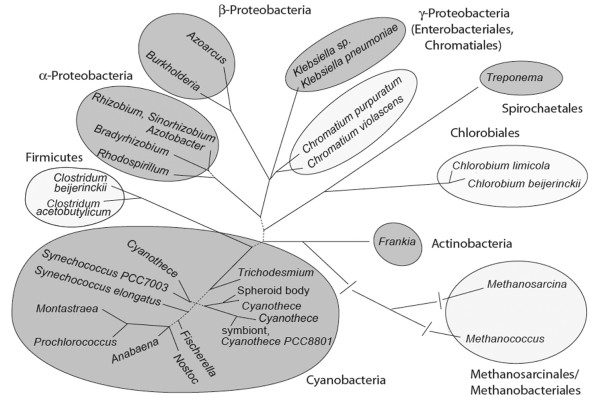
**Phylogenetic affinities of symbiotic and non-symbiotic nitrogen fixing bacteria**. Different divisions of nitrogen fixing bacteria (classes/orders for proteobacteria) are indicated. Groups containing symbiotic species are marked with grey, non-symbiotic groups with white ellipses. Further information on nitrogen-fixing bacteria, different interactions, hosts and localisation of symbionts is provided in the text and summarised in table 1. Branches receiving less than 70% non-parametric bootstrap support in analyses of ingroup taxa (428 base positions) are dotted.

The ability to fix molecular nitrogen is a widespread characteristic of prokaryotic cells, being established among various groups of bacteria including some archaea [18,19]. The distribution of BNF among archaea and eubacteria indicates that nitrogen fixation is an ancient innovation [15,20,21], which developed early in the evolution of microbial life on earth. Within the eubacteria, nitrogen fixation has been described for members of the proteobacteria, cyanobacteria, actinobacteria, spirochaetes, clostridiales, purple-sulfur (Chromatiales) and green-sulfur (Chlorobiales) bacteria (Figure 2). However, only some of these diazotrophic bacteria are known to interact with eukaryotes symbiotically (Figure 2, Table 1).

A diversity of eukaryotic partner organisms (animals, fungi, plants and protists) from different environments is involved in symbioses with nitrogen fixing bacteria (Table 1). The kind of these nitrogen fixing symbioses range from rather loose, temporary and non-specific contacts to stable and permanent interactions, the latter ones often characterised by morphological and/or physiological modifications of one or both partners and also the vertical transmission of symbionts to the next host generation. Symbionts can reside either extracellularly in more or less close association to their hosts or exist as endosymbionts intracellularly within host cells. Among these associations an interaction is considered as obligate for one partner if it is not able to survive outside the symbiotic association. In the case of symbiotic bacteria, an obligate status is often accompanied by deleterious genome evolution, e.g. the loss of genes whose products are no longer required for the new host-dependent lifestyle [22,23] whereas non-obligate (facultative) symbionts retain their autonomy and are indistinguishable from their free-living forms with respect to gene content. Numerous manifestations of symbiotic interactions between nitrogen-fixing bacteria and their hosts are known and they reflect considerable diversity and complexity. The following sections provide an overview of main types of associations and their characteristics.

**Table 1:** Free-living and symbiotic nitrogen fixing bacteria

	**Division**	**Selected species**	**Selected symbiotic members**	**Eucaryotic hosts**	**Localisation within host**	**References**
**archeae**	Methanosarcinales	*Methanosarcina *sp.	n.d.	n.d.	---	[18]
	Methanobacteriales	*Methanothermobacter *sp.	n.d.	n.d.	---	[19,20]
		*Methanobacterium *sp.				
**bacteria**	Cyanobacteria	*Nostoc *sp.	*Nostoc *sp.	Bryophytes (e.g. hornworts)	extracellular (within cavities of the gametophyte)	[79]
		*Anabaena *sp.	*Anabaena *sp.	Pteridophytes (*Azolla*)	extracellular (within cavities of the dorsal leaves)	[80,85,88]
		*Trichodesmium *sp.	*Cyanothece *sp.	Gymnosperms (cycads)	extracellular (within coralloid roots)	[81]
		*Synechococcus *sp.		Angiosperms (*Gunnera*)	intracellular (within cells of the stem gland)	[87]
		*Cyanothece *sp.		Fungi (cyanolichens)	extracellular (in cephalodia or in the thallus)	[50,51]
				Diatoms (*R. gibba*)	intracellular	[97,102]
				Sponges (*Dysidea *spp.)	extracellular	[30,31]
	Actinobacteria	*Frankia *sp.	*Frankia *sp.	Actinorhizal plants	root-nodules	[57]
	Proteobacteria	α: *Sinorhizobium *sp., *Mesorhizobium *sp.	*Bradyrhizobium *sp.	Legumes	intracellular (in root-nodules)	[63,64,68]
		β: *Azoarcus *sp., *Burkholderia *sp.	*Rhizobium *sp.	Legumes	intracellular (in root-nodules)	
		γ: *Azotobacter *sp., *Pseudomonas *sp.	*Sinorhizobium *sp.	Legumes	intracellular (in root-nodules)	
		*Klebsiella pneumoniae, Erwinia *sp.	*Azorhizobium *sp.	Legumes	intracellular (in root-nodules)	
		δ: *Gloeobacter *sp., *Desulfovibrio *sp.	*Burkholderia *sp.	AM fungi	intracellular	[54]
			*Azospirillum *sp.	Grasses/nonleguminous crops	extracellular (w/o nodulation)	[76]
			*Azoarcus *sp.	Grasses/nonleguminous crops	inter- and intracellular (w/o nodulation)	[77]
			*Klebsiella pneumoniae*	nonleguminous crops	extracellular	[107]
	Firmicutes (Clostridia)	*Clostridium *sp.	n.d.	n.d.	---	[108]
	Bacteroidetes/Chlorobiales	*Chlorobium sp*.	n.d.	n.d.	---	[109]
	Spirochaetales	*Treponema *sp.	*Treponema *ZAS-9	Termites	extracellular (in the hindgut)	[43]
	Chloroflexi	*Dehalococcoides *sp.	n.d.	n.d.	---	[110]

Overview of nitrogen fixing bacteria, including selected symbiotic interactions, possible host organisms and symbiont localisation. Details of the individual symbiotic associations are described in the text. n.d.: not detected

## Results and Discussions

### Symbioses of nitrogen fixing bacteria with sponges, corals and insects (invertebrates)

Marine sponges (Porifera) are evolutionary primordial invertebrates, which can harbour a variety of extra- and intracellular bacteria or bacterial communities [24-26]. However, the symbiotic character of these associations is well defined only in a few cases [27]. Symbioses with sponges have been described for many different groups of cyanobacteria [28], where the symbionts seem to provide their hosts with organic carbon, nitrogen or secondary metabolites [27,29]. This might also be the case for the filamentous cyanobacterium *Oscillatoria spongeliae*, which is found to be host-specific in *Dysidea *spp. [30]. Cyanobacterial symbionts of *Chondrilla australiensi*s are thought to be vertically transmitted [31,32], but an obligate status for these interactions has yet to be tested rigorously.

Corals in general are partners of endosymbiotic dinoflagellates (zooxanthellae), which provide photosynthetically derived carbon to their animal hosts [33], but nitrogen fixation by cyanobacteria is also a well-known feature of coral reefs and coral communities [34-36]. The metazoan coral *Montastraea cavernosa *is an example of a host harbouring symbiotic cyanobacteria [37]. In the *Montastraea *endosymbiosis, two symbiotic organisms, the zooxanthellae and cyanobacteria, share the same host compartment. Here, the nitrogen fixation by the cyanobacteria might be facilitated by the host providing energy rich compounds. If so, this would indicate a high degree of specificity association between all three partners [31].

Also higher invertebrates benefit from the metabolic capacities of nitrogen-fixing bacteria. The hindgut of wood-feeding termites is colonised by flagellate protozoa [38,39], which facilitate digestion of lignocellulose [40]. The carbon-rich but nitrogen-poor nature of the termite diet requires nitrogen from other sources [41]. This is thought to be provided by intracellular bacteria associated with termite gut flagellates, such as *Trichonympha agilis *in *Reticulitermes santonensi *[42]. These are examples of permanent endosymbionts placed phylogenetically in a new phylum endomicrobia [42]. Interestingly, although the endomicrobia are symbionts of the flagellate protists rather than the termites, they might best be considered as animal endosymbiotic associations. More recently, free-living spirochetes of the termite hindgut have also been revealed to fix molecular nitrogen and provide their host with nitrogen metabolites [43]. A further interaction has also been identified in *Tetraponera *ants, which harbour a subset of different bacteria in a special organ ("bacterial pouch"), among them relatives of *Rhizobium*, *Pseudomonas *and *Burkholderia *[44]. However, although these symbionts are related to nitrogen fixing and/or root-nodule associated bacteria, it is only speculated that the insect host benefits from fixation of molecular nitrogen. More likely, nitrogenous waste secreted by the host is metabolised and recycled by the bacteria. This is also indicated by the high amount of Malphigian tubules in the pouch, which transport nitrogenous waste. Nevertheless, nitrogen fixing activity of the symbiotic bacteria of *Tetraponera *cannot be excluded as a possibility. The diverse symbiotic interactions between nitrogen fixing bacteria and insects described so far share some common characteristics.

These symbionts often inhabit specialised organs or regions of the host. This localisation in turn provides an optimal environment for their activity, without symbionts needing to reside inside host cells. This is in contrast to other well-known bacterial interactions with insects, like the *Buchnera *symbiosis [45]. Here, the symbionts reside within specialised host cells and show a remarkable degree of adaptation leading to an obligate and permanent level of interaction. One prerequisite for such co-evolution of both partners is stable vertical transmission of symbionts which usually takes place maternally, via infection of eggs or larvae [45,46]. In contrast to endosymbionts, stable integration and transmission of gut and cavity symbionts seems to be challenging as they are more vulnerable for replacement by other mircobes. Ants and termites are colony organised insects and transmission of extracellular symbionts could take place horizontally via close contact of different individuals or via feeding of larvae by infected workers. However, reproduction of social insects is accomplished only by few individuals, thus vertical transmission from queens to the offspring is necessary for the foundation of new colonies. Phylogenetic analyses of the gut microbiota of termites indicate symbiont-host coevolution based on vertical transmission in combination with frequent horizontal exchange between congeneric species [47,48]. Consequently, the special social lifestyle of termites and ants might be one prerequisite for the establishment of stable vertical transmission and cospeciation of extracellular symbionts in these lineages.

### Symbioses of nitrogen fixing bacteria with fungi: cyanolichens and symbionts of arbuscular mycorrhizal fungi

In lichen symbioses, a fungal partner (mycobiont) is associated with an extracellular photobiont. The latter are mostly different photosynthetic algae, but cyanobacteria also occur as photobionts in lichens, either alone (bipartite symbiosis) or in combination with algae (tripartite symbiosis) [49]. The benefit to the photobiontic partner is not fully understood, but it might include the provision of water, minerals, protection from predators and UV damage [50]. The advantage for the fungal partner is the provision of photosynthesis-derived carbon metabolites from the photobiont. Cyanobacteria (cyanobionts) provide, in addition to carbon, fixed nitrogen to their hosts. The importance of molecular nitrogen fixation is reflected in the physiological and morphological adaptations of lichen-associated cyanobacteria. These include an increased number of nitrogen-fixing heterocysts in symbiotic *Nostoc *sp. compared to free-living filaments. A further adaptation is found in tripartite symbioses where the cyanobacteria are concentrated in special areas called cephalodias, where they fix nitrogen and are protected from high oxygen concentrations. In these tripartite symbioses, photosynthesis is restricted to the algal photobionts, and these supply the other partners with fixed carbon compounds [51]. The fact that most cyanobionts are not vertically transmitted and are also found as free-living organisms indicates that they are not obligate symbionts, and thus not dependent on host metabolism. Nevertheless, the morphological characters of lichens suggest a high degree of coevolutionary adaptation of all participants. Although commonly considered a mutualistic interaction, some hypotheses propose that lichen symbioses are a form of parasitism [50]. Even so, the ecological and evolutionary success of lichens suggests mutual benefit is characteristic for the association.

The arbuscular mycorrhizal (AM) symbiosis between fungi and plant roots is the most common of this type of interaction in the rizosphere [52]. The fungus supplies the plant with water and nutrients such as phosphate, while the plant provides the fungus with photosynthetically produced carbohydrates. The AM fungus *Gigaspora margarita *harbours intracellular bacteria from the genus *Burkholderia *[53,54], which supply the fungus with fixed nitrogen. However, the extent of physiological adaptation or reduction of these endosymbionts leading to an obligate status of interaction has yet to be determined.

A further symbiosis, discovered in the Spessart-mountains (Germany), was identified by analysing the fungus *Geosiphon pyriformis*, related to AM fungi [55]. At the hyphal tips of this fungus, unicellular multinucleated "bladders" develop, which harbour *Nostoc punctiforme*. It has been shown that these bladders fix CO_2_, which may be the major contribution of the cyanobacterium to the symbiosis. The symbiont also forms heterocysts, suggesting that nitrogen is fixed as well [56]. However, as these heterocysts are somewhat similar to those of free-living relatives of this *Nostoc *strain, nitrogen fixation may only serve the needs of the symbiont itself.

### Symbioses of nitrogen fixing bacteria with plants

Interactions of bacteria with various groups of plants are the most common symbiotic association for nitrogen assimilation. A multiplicity of bacteria with different physiological backgrounds are involved in these associations, including gram-negative proteobacteria like *Rhizobia sp*. and *Burkholderia sp*., gram-positive *Frankia *sp. [57] and filamentous or unicellular cyanobacteria [58]. The physiological and morphological characteristics of these symbioses range from extracellular communities to highly adapted interfaces within special organs or compartments.

**Figure 3:**
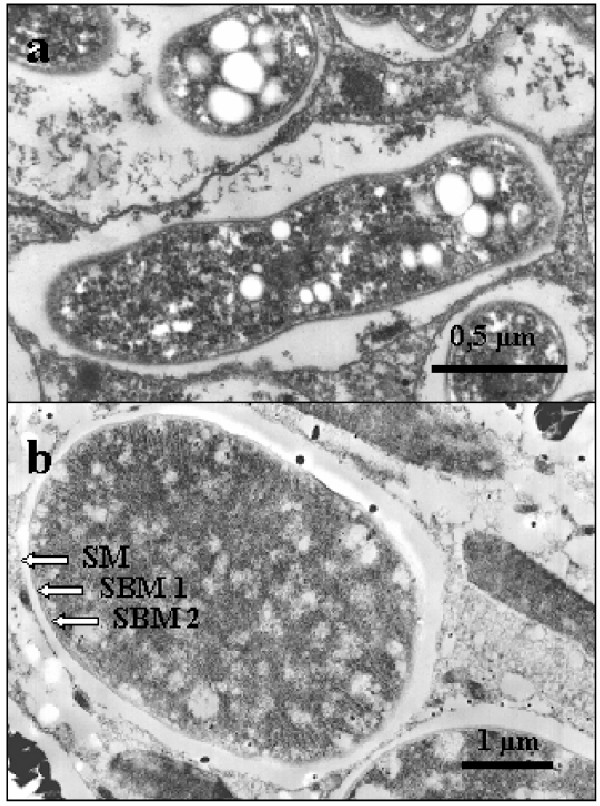
**Endosymbionts adapted for molecular nitrogen fixation **a) A *Bradyrhizobium *sp. bacteroid in a root-nodule of *Glycine max *(soybean). b) A Spheroid body of the diatom *Rhopalodia gibba*. SM: Symbiontophoric membrane SBM: Spheroid body membrane.

The mutualistic symbioses between various non-photosynthetic proteobacteria of the order Rhizobiales with plants of the orders Fabales, Fagales, Curcurbitales and Rosales are the most extensively studied interactions between bacteria and plants [59]. The rhizobia-legume symbiosis is characterised by typical root-nodule structures of the plant host, which are colonised by the endosymbiotic rhizobia, so-called bacteroids [60]. The nodulated plant roots supply the bacteria with energy-rich carbon compounds and obtain fixed nitrogen by the bacteroids in return. The nodule formation is a highly regulated and complex process driven by both partners. Free-living rhizobia enter the plant root epidermis and induce nodule formation by reprogramming root cortical cells. Of special importance for the establishment of the symbiosis are flavonoids secreted by the plant partner [61] and the subsequent induction of bacterial nodulation (*nod*) genes [62]. The Nod-factors play a role in the formation of the nodule, a complex structure optimised for the requirements of both partners [63,64]. Analysis of root epidermal infection and the underlying signal transduction pathways [65-67] indicate that Nod-factors may have evolved following recruitment of pathways, which developed in a phylogenetically more ancient arbuscular mycorrhiza symbiosis [68,69]. In the nodule, bacteroids reside within parenchym cells, where they are localised in membrane bound vesicles (Figure 3a) [70]. Nitrogenase activity is ensured by the spatial separation of the bacteroids inside the nodule structure and special oxygen-scavenging leghemoglobin that is synthesised in the nodules [71]. An interesting feature of rhizobia is that nitrogen fixation is restricted to symbiotic bacteroids, whereas free-living bacteria do not express nitrogenase [72]. Although the rhizobia-legume symbiosis is a highly adapted and regulated interaction it can not be termed permanent or obligate. Both partners can live and propagate autonomously, and each host generation has to be populated by a new strain of free-living rhizobia.

Rhizobia-legume symbioses are not the only root-nodule forming interactions of bacteria and plants. Actinobacteria of the genus *Frankia spp*. are known to develop nodules for nitrogen fixation in various families and orders of angiosperms known as actinorhizal plants [73]. Free-living *Frankia *is characterised by a unique morphology, including three structural forms, hypha, sporangium and vesicle, the latter one being a compartment for nitrogen fixation. Although functionally analogous, *Frankia *nodules differ from those in rhizobia-legume interactions in development and morphology [74]. In contrast to rhizobia all *Frankia *strains are also capable of fixing molecular nitrogen as free-living bacteria [75]. The appearance of the *Frankia*-symbiosis as a nodulation dependent interaction emphasises the adaptation of both partners. Other plants, including important economic crops like *Zea mays *and *Oryza sativa *have established associations with different nitrogen-fixing bacteria, including *Azospirillum *[76] and *Azoarcus *[77]. However, such symbioses have never been found to result in nodule formation.

In addition, nitrogen fixing cyanobacteria are also often found interacting with plant partners. For example, symbioses of filamentous heterocyst-forming *Nostoc *sp. have been reported for bryophytes, pteridophytes (*Azolla*), gymnosperms (cycads) and angiopsperms (*Gunnera*) [78-81]. In all plant hosts, with the exception of *Gunnera*, symbiotic *Nostoc *filaments are localised extracellularly in different locations depending on the host species. In bryophytes, like hornworts, the cyanobacteria are found within cavities of the gametophyte [79], whereas an *Azolla *sp. harbours the bacterial partners in cavities of the dorsal photosynthetic parts of the leaves [80]. In cycad-cyanobacterial associations the symbionts are limited to specialised coralloid roots where they reside in the cortical cyanobacterial zone [81]. More specialised is the mutualistic intracellular *Gunnera*-*Nostoc *symbiosis. Here the process begins with invasion of the petiole glands, followed by intracellular establishment within the meristematic cells of this tissue [60,78].

The symbioses of cyanobacteria with their plant partners differ remarkably from the rhizobia-legume interactions. First, cyanobacteria show a broad host range and thus differ from rhizobia or *Frankia *sp., which are limited to legumes or angiosperms, respectively. In addition, cyanobacteria do not induce the formation of highly specialised structures like root-nodules after colonisation of the host but reside in plant structures known as symbiotic cavities [82], which also exist without symbiosis. The lack of nodule-like organs can be explained by the fact that heterocyst forming cyanobacteria also fix nitrogen as free-living cells and therefore do not need a special environment for N_2_-fixation in symbiosis. This makes them distinct from rhizobia, which only fix nitrogen in the protective environment of the nodule. Although symbiotic cavities do not display the close and highly regulated interface of a legume-nodule they are nevertheless regions that exhibit adaptations for symbiosis. A common specialisation in occupied symbiotic cavities of plant hosts is the elaboration of elongated cells to improve nutrient exchange [83] and the production of mucilage-exopolysaccharides for water storage or as nutrient reserve (e.g. [84,85]). The infection process is controlled via the production of hormogenium-inducing factors by the host plant, resulting in the development of vegetative cyanobacterial filaments (hormogonia), important for host colonisation [86,87]. The main adaptations to the symbiotic lifestyle found in the bacterial partners concern changes of morphology and physiology. These include a remarkable increase of heterocysts in symbiotic *Nostoc*, and higher rates of N_2 _fixation compared to those of free-living cells. In addition, photosynthesis of symbiotic cyanobacteria is depressed in various associations to avoid competition between symbionts and host for CO_2 _and light [86].

In conclusion, different adaptations are found in cyanobacterial-plant interactions but they are not as specific and highly regulated as the complex nodule-forming symbioses. A common feature of all bacteria plant symbioses is their non-obligate, non-permanent character, including a lack of vertical transmission of symbionts to the next host generation. An exception might be the *Nostoc*-*Azolla *symbiosis, where cyanobacterial homogenia are transmitted via megaspores [88].

### Symbioses of nitrogen fixing bacteria with protists

Symbioses of bacteria with unicellular eukaryotes are exceptional as they involve the whole host rather than specialised parts of the host organism. Also these intracellular symbionts require a high degree of regulation and adaptation to maintain the mutualistic relationship. This feature, in conjunction with vertical transmission, suggests that co-evolution and dependence of partners is sufficiently advanced to regard the relationship as unification of two single organisms. The mitochondria and plastids of recent eukaryotes are extreme examples of this kind of association [89,90]. Cyanobacteria have also been detected in intracellular association with an euglenoid flagellate [91], heterotrophic dinoflagellates [92-94], a filose amoeba [95], diatoms [96,97] and, extracellularly, with some protists, e.g. diatoms [98]. Only rarely has the nitrogen fixing activity of the prokaryotic partner been demonstrated in these symbioses (e.g. [99]). In the next paragraph the range of symbiotic associations between cyanobacteria and protists is described in a progression of interactions from temporary to permanent. As such, these symbioses provide an opportunity to investigate the cellular changes that may accompany the evolutionary transition from extracellular symbiont to intracellular endosymbiont and cell organelle.

*Petalomonas sphagnophila *is an apoplastic euglenoid that harbours endosymbiotic *Synechocystis *species [91]. The cyanobacteria occur inside a perialgal vacuole and remain alive for several weeks, before they are metabolised, so that they must be regarded as temporary endosymbiotic cell inclusions. These intracellular cyanobacteria are thus reminiscent of kleptochloroplasts found in some heterotrophic dinoflagellates, marine snails, foraminifera and ciliates. These associations can be understood as a mechanism for the temporary separation of ingested and digested prey [92-94,100]. However, in all well-documented cases of kleptochloroplastic interactions, only the plastid or the plastid together with surrounding cell compartments (never the whole cell) is incorporated as a kleptochloroplast by the host. In contrast, the cyanobacteria of *P. sphagmophila *are not disintegrated during their internalisation by the euglenoid [91]. Symbiont integrity is therefore likely to be a prerequisite for the functioning of the cyanobacterial nitrogen fixing machinery. The enslaved cyanobacteria may also provide energy-rich C-compounds or, as suggested for other symbiotic interactions, vitamin B12 production to it host [101]. These hypotheses are yet to be investigated thoroughly.

Phaeosomes are symbionts found in some representatives of the order *Dinophysiales*. They exhibit morphological characteristics of *Synechocystsis *and *Synechococcus *cells and are located either extracellularly or intracellularly [94]. In the case of intracellular cells, the symbioses seem to be permanent and the benefit of the symbiosis to the host may be efficient nitrogen fixation. However, as in the case of *P. sphagnophila*, difficulties in cultivating these strains complicate molecular characterisation of the endosymbionts. At present this problem is limiting our understanding of the potential benefits of these prokaryote/eukaryote mergers. Some filamentous cyanobacteria are known to interact with diatoms. Extracellular epibionts, endosymbionts and also symbionts positioned in the periplasmic space between the cell wall and cell membrane of the diatom are known to occur [58,98]. Electron microscopy scanning of such interactions has demonstrated a dual symbiotic nature of some symbionts. E. g. *Richelia intracellularis *has been observed to interact either as an epibiont (with *Chaetoceros spec*.) or as endosymbiont (with *Rhizosolenia clevei*) [98]. In these examples, nitrogen fixation for the benefit of the host has been demonstrated by the cultivation of the symbiont-diatom association in the absence of an external fixed nitrogen source. Nitrogen fixation is also suggested from morphological features such as the presence of heterocysts. At least in tropical environments, the production of B12 vitamins may also be a further benefit for the host [101].

### The cyanobacterial endosymbionts of the diatom *Rhopalodia gibba*

Some diatoms, including *Climacodium frauenfeldianum *and *Rhopalodia gibba*, are known to harbour permanent endosymbionts [96,97,102]. As indicated by EM investigations of *R. gibba*, these endosymbionts are intracellular and are transmitted vertically [102,103]. The endosymbionts, so-called spheroid bodies [96], are localised in the cytoplasm, and separated by a perialgal vacuole from the cytosol. Each spheroid body is surrounded by a double membrane. As additionally internal membranes are also visible, this morphotype is similar to that of cyanobacteria (Figure 3b). 16S rDNA sequences have been amplified from an environmental sample of *C. frauenfeldianum *[97] and from isolated spheroid bodies of *R. gibba *[102]. Phylogenetic analysis groups these sequences together with free-living cyanobacteria of the genus *Cyanothece *(Figure 2). This robust grouping is also evidenced from phylogenetic analysis of a nitrogenase subunit gene, isolated from *R. gibbas*'s spheroid body [102]. In phylogenetic reconstructions of both genes, the branch lengths separating free-living cyanobacteria and the cell inclusions of *C. frauenfeldianum *and *R. gibba *are very short, indicating that origins of the protist symbioses are relatively recent. This is unlike the situation for plastids and extant cyanobacteria, which have an ancient phylogenetic relationship. *Cyanothece *sp., the closest known free-living relatives of spheroid bodies and the endosymbiont of *C. frauenfeldianum*, are typical unicellular and diazotrophic cyanobacteria. To protect the nitrogenase from oxygen tension, *Cyanothece *show a strong physiological periodicity, restricting nitrogen-fixation exclusively to the dark period of growth [104]. During this period, the energy demand for N_2 _fixation is sustained by large amounts of photosynthetically derived carbohydrates, which are stored as starch particles. Nitrogen fixing activity of *R. gibba *was first indicated in the 1980s via acetylene reduction assays [99] and confirmed in latter studies [102]. Intracellular localisation of the enzymatic activity has been undertaken by scanning for protein subunits of nitrogenase [102]. Immunogold experiments have shown that the nitrogenase is localised within the diatom spheroid bodies, thereby confirming that the endosymbiont is responsible for the fixation of nitrogen. Furthermore, corresponding genes for the nitrogenase activity have also been isolated from purified spheroid bodies [102]. Interestingly, spheroid body nitrogen fixation in *R. gibba *is a strictly light dependent process. This might be the result of several adaptations to the endosymbiotic lifestyle. Spheroid bodies lack a characteristic cyanobacterial fluorescence based on photosynthetic pigments, indicating that they have lost photosynthetic activity and that energy for nitrogen fixation is supplied by the host cell. The protection of the nitrogenase enzyme complex is accomplished through the spatial separation of the two pathways, with N_2 _fixation in spheroid bodies and photosynthesis in the host plastid. The loss of photosynthetic activity of spheroid bodies is also expected to lead to the loss of autonomy resulting in an obligate endosymbiosis. This hypothesis is consistent with the observation that *R. gibba *cells are never observed without spheroid bodies and that cultivation of the endosymbionts outside the host cells has not been possible [102]. Definitive evidence is still required to determine the exact nature of symbiotic interaction and whether the spheroid body of *R. gibba *is an obligate endosymbiont, or perhaps even an unrecognised DNA-containing organelle.

## Conclusion

The ability to fix molecular nitrogen is restricted to selected bacterial species that express the nitrogenase enzyme complex. Nevertheless, various eukaryotic organisms have utilised this capacity by establishing symbiotic interactions with nitrogen fixing bacteria. In these associations, fixed nitrogen is provided to the hosts, thereby enabling them to colonise environments where the supply of bound nitrogen is limited. In mutualistic symbioses, bacterial symbionts benefit from these associations, e.g. by protection against predators or by being provided with host metabolites. Symbioses for molecular nitrogen fixation can be found in many different habitats, with host organisms including all crown groups of eukaryotic life. Although all partnerships are based on the same enzymatic reaction, the diverse associations differ with respect to the physiological and morphological features that characterise the interconnection of partners. Such features include the development of special host organs for optimal performance of bacterial symbionts, adaptations in host and symbiont metabolism, and the intracellular establishment of bacteria within the host.

Close associations involving multiple adaptations and co-evolution between partners can result in permanent and obligate relationships, whereby the bacterial symbiont is stably integrated into the host system, and vertically transmitted across generations. These close interactions are mainly found in intracellular symbioses, where free-living bacteria reside within the cells of the host organism. These are similar to organelles of eukaryotes, such as mitochondria and plastids, which both derived from symbiotic interactions and where continuous adaptation and co-evolution lead to a fusion of two distinct organisms [3,4]. In both cases, the metabolic capacity of the bacterial symbiont was the driving force for maintenance and evolutionary establishment, resulting in an inseparable merger of host and symbiont. The same basis of interaction applies for molecular nitrogen fixation, where eukaryotic hosts benefit from the unique metabolic capacity of special bacteria, leading to various symbiotic interactions with different specifications. In particular, bacteria interacting with protists, like the spheroid bodies of *R. gibba*, might serve in the future as important model systems for investigating the establishment of molecular nitrogen fixation in eukaryotic hosts. The detailed study of this interaction will thus provide a great opportunity to understand the complex mechanisms underlying the evolution of obligate endosymbionts and organelles.

## Methods

### Phylogenetic analysis

Tree construction for Figure 2: 16S rDNA gene tree built using PhyML [105] assuming the optimal substitution model determined by ModelTest [106]. For eubacterial sequences this was a K81 + I + G model, and for eubacteria and archaea a GTR + I + Γ model.

## Authors' contributions

CK and UGM conceived of this review and drafted the manuscript. CV and PL participated in preparing the final manuscript. PL performed the phylogenetic analyses. All authors read and approved the final manuscript.
